# Mainstreaming biodiversity: A review of national strategies

**DOI:** 10.1016/j.biocon.2019.04.016

**Published:** 2019-07

**Authors:** Penelope R. Whitehorn, Laetitia M. Navarro, Matthias Schröter, Miguel Fernandez, Xavier Rotllan-Puig, Alexandra Marques

**Affiliations:** a Karlsruhe Institute of Technology KIT, Institute of Meteorology and Climate Research Atmospheric Environmental Research (IMK-IFU), Kreuzeckbahnstraße 19, 82467 Garmisch-Partenkirchen, Germany; b German Centre for Integrative Biodiversity Research (iDiv), Halle-Jena-Leipzig, Deutscher Platz 5e, 04103 Leipzig, Germany; c Institute of Biology, Martin Luther University Halle-Wittenberg, Am Kirchtor 1, 06108 Halle (Saale), Germany; d UFZ – Helmholtz Centre for Environmental Research, Department of Ecosystem Services, Department of Computational Landscape Ecology, Permoserstr. 15, 04318 Leipzig, Germany; e NatureServe, 4600 N. Fairfax Dr., Arlington, VA 22203, USA; f Smithsonian-Mason School of Conservation, Department of Environmental Science & Policy, George Mason University, 400 University Drive, Fairfax, VA 22030, USA; g ASTER-Projects, Barri Reboll 9 1r, 08694 Guardiola de Berguedà, Barcelona, Spain; h European Commission, Joint Research Centre (JRC), Ispra, Italy

**Keywords:** Biodiversity loss, Mainstreaming, NBSAPs, Aichi targets, Economic sectors

## Abstract

Biodiversity is suffering dramatic declines across the globe, threatening the ability of ecosystems to provide the services on which humanity depends. Mainstreaming biodiversity into the plans, strategies and policies of different economic sectors is key to reversing these declines. The importance of this mainstreaming is recognized by the Convention on Biological Diversity (CBD) and its Aichi targets. Individual countries can implement the goals of the CBD through their National Biodiversity Strategies and Action Plans (NBSAPs), which aim to, inter alia, support the mainstreaming of biodiversity into the policies of key economic sectors, such as agriculture, forestry and fisheries. This paper investigates the performance of countries at incorporating biodiversity mainstreaming into their post-2010 NBSAPs. We conduct a large-scale review of 144 NBSAPs against five criteria and calculate a national-level indicator for comparing levels of mainstreaming among countries. Our results show that developing countries, particularly those in Africa, have higher scores, indicating that they have a higher awareness of the importance of biodiversity mainstreaming. Developing nations were also more likely to involve a greater range of stakeholders in the NBSAP development process, whilst developed nations were less likely to give specific details about the monetary contributions of biodiversity to their economies. Overall, our findings suggest that biodiversity mainstreaming remains a challenge across much of the world, but that progress in some areas can provide direction and momentum in the future.

## 1 Introduction

The benefits that biodiversity provide to human well-being are manifold (Díaz et al., 2018). Biodiversity underpins ecological processes that form the basis of a plethora of ecosystem services including food provision, clean drinking water, regulation of pests and diseases, maintenance of soil fertility and non-material benefits such as opportunities for aesthetic appreciation and recreation (Harrison et al., 2014). However, humanity has transformed the majority of the planet's surface to meet its immediate needs (Foley et al., 2005; IPBES, 2018a; IPBES, 2018b). The resulting global change has been causing serious declines in the distribution and abundance of species, the composition of communities and the ecological functions within ecosystems (Pereira et al., 2012; Tittensor et al., 2014). This loss of biodiversity threatens the ability of the living planet system to provide the services on which humanity so depends (Cardinale et al., 2012; IPBES, 2018a).

Addressing direct pressures and causes is imperative to halt these global biodiversity losses. This has become the global mission of the Convention on Biological Diversity (CBD). This international treaty, which became effective in December 1993 and now has 196 signatory countries, aims to ensure the conservation and sustainable use of biodiversity. The CBD recognizes “*that the objectives of the Convention would be impossible to meet until consideration of biodiversity is fully integrated into other sectors*” (SCBD, 2005). This integration is key, as the main direct pressures on biodiversity originate within economic sectors such as agriculture, forestry and fisheries (Kok et al., 2014; Kok et al., 2018). This process of integrating biodiversity concerns and actions related to biodiversity conservation into different economic sectors and development plans is referred to as “biodiversity mainstreaming” (cf. GEF, UNEP and CBD, 2007; IIED and UNEP-WCMC, 2017; SCBD, 2014).

Biodiversity mainstreaming has become a major global challenge (Karlsson-Vinkhuyzen et al., 2018). This endeavor was reinforced by the CBD's Strategic Plan for Biodiversity 2011–2020 and its Aichi biodiversity targets, which were adopted at the 10th meeting of the Conference of Parties (COP) in 2010. Specifically, countries agreed to “address the underlying causes of biodiversity loss by mainstreaming biodiversity across government and society” (CBD, 2010), and defined their own sets of national targets following the Aichi targets framework. This ambitious global goal can be reached by each country in primarily two ways, encapsulated by two Aichi targets. First, Target 2 calls for biodiversity values to be integrated into development strategies and planning processes. Achieving this target means that biodiversity is considered across all sectors, at both national and local levels, thus enabling a proactive and preventative attitude towards its conservation and ideally a shared sense of responsibility (Persson et al., 2018). Second, Target 17 requires the development of National Biodiversity Strategies and Action Plans (NBSAPs), which are the primary instrument for implementing the Convention at the national level and are updated to explicitly address the 20 Aichi Biodiversity Targets (of the 190 Parties that have prepared NBSAPs, 148 had revised them at least once by September 2018, CBD, 2018a). These updated plans aim to feed into concrete, specific policy instruments, hence supporting the mainstreaming of biodiversity into the activities of sectors that impact it the most (CBD, 2010; Kok et al., 2010). The importance of mainstreaming is such that, if effectively implemented, these two targets could have a positive effect on the achievement of all the remaining Aichi targets (Marques et al., 2014). The Aichi targets 5 to 10, under Strategic Goal B, concerning the sustainable use of biodiversity, should be highly influenced by mainstreaming actions but they can also promote mainstreaming per se if sustainable managements plans recognize the value of biodiversity (OECD, 2018).

More recently, the CBD has reiterated the importance of biodiversity mainstreaming with the Cancun Declaration (CBD, 2016), which was adopted at the 13th COP meeting in 2016. This declaration recognizes that biodiversity protection must involve a range of different governmental and economic sectors. More than 190 countries have pledged to increase efforts to integrate biodiversity into policies of their forestry, fisheries, tourism and agriculture sectors. This has since been expanded with the Sharm El-Sheikh Declaration (CBD, 2018b), adopted at the 14th COP meeting in 2018, which adds that mainstreaming biodiversity should also occur in the energy, infrastructure, manufacturing and processing sectors.

Despite this clear global objective and its declination into national obligation, the road to biodiversity mainstreaming has thus far not been a smooth one. Key actors in relevant economic sectors still consider biodiversity to be distant from their key interests and countries developing their NBSAPs have found it challenging to mainstream biodiversity into economic development (Prip et al., 2010; Leadley et al., 2014; Karlsson-Vinkhuyzen et al., 2017). Previous analyses of the fourth and fifth national reports, which present the progress made on the implementation of the Convention's objectives, revealed that the key barriers to biodiversity mainstreaming are: short-term economic gains by the primary production sector, fragmented decision making, and limited communication among stakeholders (Chandra and Idrisova, 2011; Leadley et al., 2014). This limited communication is likely due to the lack of involvement in the NBSAP development process, as the preliminary post-2010 NBSAP assessments showed that neither the private sector nor members from civil society were consistently consulted (Pisupati and Prip, 2015).

Limited overall efforts at mainstreaming biodiversity have also been found in an investigation of four case studies in agriculture, agro-forestry and fisheries, exemplifying transnational governance of both land and water (Karlsson-Vinkhuyzen et al., 2018). Some institutional levers were identified that aided mainstreaming, such as co-management structures in mangroves in Vietnam involving fishermen and local authorities, and trust building in certified palm oil and marine fisheries. But motivational and means barriers to mainstreaming were more common, such as short term visions and a severe lack of financial resources, time and knowledge. This lack of means has traditionally hampered and undermined conservation programmes in developing counties and transitional economies (Chandra and Idrisova, 2011).

However, the outlook is not entirely bleak and there are mainstreaming success stories. Huntley (2014) has identified positive mainstreaming in South Africa and Costa Rica, helped by the extremely high levels of biodiversity in each country, which, despite high levels of threat, has led to high interest and support from donors. This financial support has been further assisted by the democratic and transparent governance systems that provide security and longevity to mainstreaming investments. This demonstrates how mainstreaming strategies need to be accompanied by nature protection policies and political support to be truly effective (Karlsson-Vinkhuyzen et al., 2017). Another success story comes from the global fisheries sector. Friedman et al. (2018) find that the ‘architecture for the mainstreaming of biodiversity’ has developed considerably over the last two decades across international, national and regional frameworks. This has developed because of strengthened communication and discovered common ground between the fisheries and biodiversity conservation communities. Improved communication, which has enabled cross-sectoral institutional collaborations on policies and actions, has also proved key to the success stories that have emerged from the Mainstreaming Biodiversity and Development project, facilitated by IIEC, UNEP-WCMC (2015).

Despite this recent growth in research on mainstreaming biodiversity, global patterns on how countries have incorporated it into their updated post-2010 NBSAPs remain unclear. The OECD conducted a small-scale review of 16 NBSAPs and found a variable picture of how countries were mainstreaming biodiversity (OECD, 2018). The CBD conducted an internal analysis of NBSAPs received after adoption of the Strategic Plan for Biodiversity 2011–2020 and found very low levels of mainstreaming (CBD, 2018a). Only 28% of 159 Parties had conducted valuation studies of biodiversity, 20% state that biodiversity has been integrated into national development plans and 13% mention the integration of NBSAPs with sustainable development plans.

The aim of this paper is to build on the analyses mentioned above and conduct a large-scale review of how post-2010 NBSAPs mainstream biodiversity into relevant economic sectors. This will allow us to reach a greater understanding of the extent to which the value of biodiversity is prioritized at a national level and how the NBSAP-process is performing as an instrument to mainstream biodiversity.

## 2 Methods

The rationale behind our analysis is that NBSAPs act as inputs to other sectoral policies, influencing and altering their approach to biodiversity. Integration of biodiversity into sectoral policies would then ideally promote a change in behavior that in turn would result in an improved biodiversity state. We assume that if biodiversity is clearly mainstreamed into the economic sectors in the NBSAPs, then it would potentially be more effective in influencing sectoral policies.

To understand the extent to which biodiversity is being mainstreamed across economic sectors we performed a review of the NBSAPs released before the end of October 2017 (https://www.cbd.int/nbsap/about/latest/). We restricted our review to NBSAPs written in either English, Spanish or French. A total of 144 NBSAPs were reviewed against five questions, developed on the work from IIED (IIED, 2015) (Table 1), using a standardized set of keywords (Table S1) to minimize the role of reviewer interpretation. The economic development of each country analyzed was defined as either ‘Developed’ or ‘Developing’ using the UN classifications (https://unstats.un.org/unsd/methodology/m49/). See Table S2 for details of the individual countries analyzed.

**Table 1:** Questions used to review the NBSAPs and the possible maximum scores for each question. For questions 1 and 3 to 5 each answer option (in brackets) yields a score of 1, for question 2 a specific answer yields a score of 2 and a vague answer a score of 1.

Questions	Maximum score
1. Which actors have been involved in the development of the NBSAP? (*public*, *private*, *civil society*, *other*)	4
2. Are there references to the (potential) contribution of biodiversity or ecosystem services to the national economy? (*yes*-*specific*, *yes*-*vague*)	2
3. Is it discussed if biodiversity loss threatens the outcomes of particular sectors? (*agriculture*, *forestry*, *fisheries*, *tourism*, *water supply*, *other extractive activities*, *other*)	6
4. Is it discussed how sustainable management plans (and hence biodiversity conservation) can contribute to the improvement of the outcomes of particular sectors? (*agriculture*, *forestry*, *fisheries*, *tourism*, *water supply*, *other extractive activities*, *other*)	6
5. Is it discussed if biodiversity conservation threatens the outcomes of particular sectors? (*agriculture*, *forestry*, *fisheries*, *tourism*, *water supply*, *other extractive activities*, *other*)	6

### 2.1 Justification of questions

Question 1 asks whether the opinions, knowledge and/or requests of society as a whole have been considered during the development of NBSAPs. A policy instrument, such as an NBSAP, is more likely to be effective if it takes into account the concerns of all actors of society (Huntley and Redford, 2014; SCBD, 2007). The categories for society used in this review were public, private and civil society and finally ‘other’, which includes indigenous people as well as members of the public.

Countries that are aware of the beneficial contributions that biodiversity and ecosystem services make to its development are more likely to effectively mainstream biodiversity (Huntley and Redford, 2014). Therefore, question 2 examines whether the (monetary) value of biodiversity or ecosystem services is acknowledged in the NBSAPs, and whether these references are specific, detailing the actual values of contributions in some quantitative way (monetary if appropriate), or simply acknowledgements that a value does exist.

Questions 3, 4 and 5 examine how NBSAPs identify the relationship between biodiversity and different production sectors. The sectors included in this review were agriculture, forestry, fisheries, tourism, water supply, other extractive activities (such as mining) and other (for example, renewable energy and infrastructure development). Question 3 aims to understand whether biodiversity loss is considered a threat to the performance of the aforementioned sectors. In principle, the identification of these relationships in the NBSAPs is a clear indication that countries recognize the need to integrate biodiversity concerns into sector planning. Question 4 seeks to understand the extent that sustainable management plans or other biodiversity conservation actions are perceived as having a positive impact on the development of particular sectors. The identification of this relationship is a positive sign for effective mainstreaming. Finally, Question 5 investigates whether there is a perceived conflict or trade-off between biodiversity conservation and the output of production sectors.

### 2.2 Scoring

We assigned a maximum score for each question, related to the possible maximum number of positive answers to the questions (detailed in Table 1). A point was added for each question whenever one of the possible answer categories was met. For instance, for question 1, if a NBSAP indicated that the public sector, private sector and civil society were involved in the development of the report, then the score was three (of a maximum of four). For questions 3 to 5 this score was capped at six (even though there are seven possible answer categories), to account for the fact that some sectors are of less importance in some countries. For example, in a country with no sea access it is likely that the fisheries sector has less importance when compared to the agricultural sector. Hence, capping the score at six prevents such countries from receiving a lower overall score simply for a geographical difference. As different people performed the reviews, it is recognized that the final score allocated for each question is based on the somewhat subjective judgment of the individual reviewer. However, differences in interpretation were minimized by using key words in the review process and the binary nature of the questions.

Following the calculation of scores for each question, an aggregated normalized score (NS) was computed for each country, which gives equal weight to all questions and has a maximum value of five:

$$\mathrm{NS}=\frac{Q1}{4}+\frac{Q2}{2}+\frac{Q3}{6}+\frac{Q4}{6}+\frac{Q5}{6}$$

These final scores provide an indicator that allows the level of biodiversity mainstreaming to be compared among countries.

## 3 Results

The normalized scores of the 144 NBSAPs reviewed ranged from 0.42 (Germany) to 4.50 (Namibia) with an overall mean of 2.45. The maximum possible score of five was not achieved by any country. The mean normalized score varied by geographical region, with Africa achieving an overall higher normalized score (mean 2.76), followed by the Americas (mean 2.66, countries analyzed in this region were mainly from Latin America and the Caribbean, Canada being the only country from North America that is party to the CBD). Asia had a mean normalized score of 2.36, Europe had a mean score of 2.15 and Oceania had the lowest mean score of 1.88 (only eight countries were analyzed in this region).

**Fig. 1:**
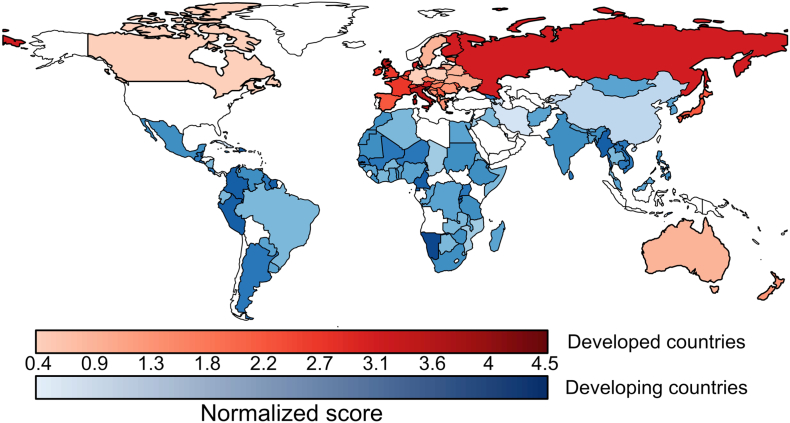
Normalized scores for each country. Red colors indicate developed nations and blue colors developing nations. Darker tones indicate higher normalized scores. Countries that were not analyzed are shown in white. (For interpretation of the references to colour in this figure legend, the reader is referred to the web version of this article.)

Developed countries, the majority of which are located in Europe, scored lower than developing countries, with an overall mean of 2.08 compared to 2.60 (see Fig. 1 for the variation within these means).

### 3.1 Stakeholder involvement (Question 1)

**Fig. 2:**
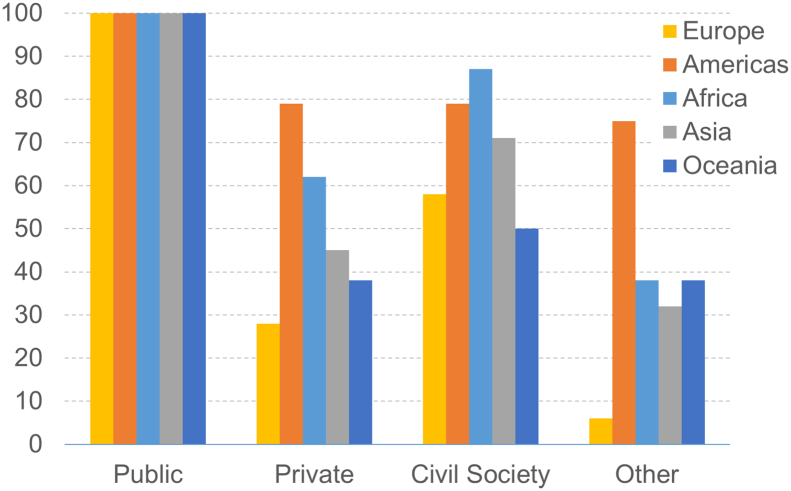
Stakeholder involvement in NBSAP development, broken down by geographic region.

In all countries analyzed, the public sector was involved in the development of the NBSAP, which is unsurprising given that national governments are responsible for this process. Civil Society was also involved in developing 72% of action plans, often through NGO's or non-profit organizations. The private sector was involved in developing 51% of action plans, whilst ‘other’ society were only consulted for 37% of the national strategies. There is variability in these figures according to geographic region (Fig. 2), with the countries of Europe (all of which have a developed economic status) showing considerably less involvement of other groups outside of the government. Overall, developing nations involved a higher percentage of stakeholders (62% private sector, 81% civil society and 46% ‘other’ society) compared to developed nations (25% private sector, 53% civil society and 5% ‘other’ society).

### 3.2 Contribution of biodiversity and ecosystem services to the economy (Question 2)

We found that the majority of NBSAPs (91%) do acknowledge the contribution of biodiversity to the national economy, and 43% of the NBSAPs provide specific details about it. For example, Seychelles' NBSAP (p. 56) highlights that biodiversity contributed to 35% of total government's revenue, 38% of national employment and 60% of gross domestic product (GDP). Peru's NBSAP (p. 27) states that 22% of the national economy is linked with biodiversity and that the legal trade of biodiversity products, which involved 46 species of native flora and fauna, represented more than 218 million US dollars in 2013.

**Fig. 3:**
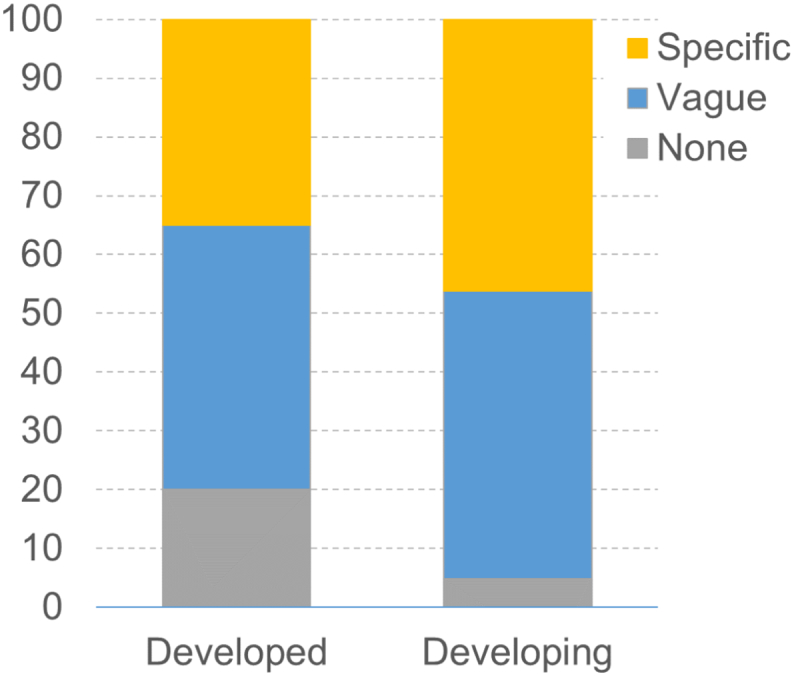
In what manner NBSAPs refer to the (potential) contribution of biodiversity and/or ecosystem services, according to the economic status. Specific = actual (monetary) value of one or more contributions are detailed; Vague = existence of value of one or more contributions are mentioned, but no actual monetary values are given.

There are, however, broad differences according to the economic development of the country (Fig. 3). Developing nations are more likely to give specific details about the (monetary) contribution of biodiversity and ecosystem services to their economy in their national strategy, whilst a higher proportion of developed nations made no acknowledgement of this contribution.

### 3.3 Contribution of biodiversity and ecosystem services to outcomes of sectors (Questions 3 to 5)

Biodiversity loss is perceived as a threat to the outcomes of production sectors (Question 3) in 85% of the NBSAPs, but in only 37% of the NBSAPs is this threat mapped to four or more production sectors. Agriculture is the most mentioned sector, followed by fisheries, forestry and water. The variation in these results by geographic region and economic development is shown in Table 2. Africa has the highest percentages for agriculture, forestry, fisheries and the availability of clean water, showing that the majority of reviewed countries recognize that biodiversity loss threatens the outcome of these sectors. For example, Niger's NBSAP (p. 37) recognizes that the loss of forest biodiversity leads to unemployment and a decrease in forest productivity. In contrast, the region where more countries identified the loss of biodiversity as a threat to the outcome of tourism was in Europe.

**Table 2:**
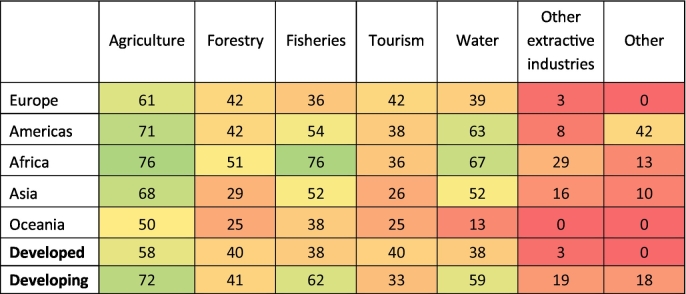
Percentage of NBSAPs, within each geographic region and economic group, identifying biodiversity loss as a threat to the outcome of each sector (Question 3). The colors are on a scale of red (0%), through yellow (50%) to green (100%).

The potential opportunities arising from sustainable management to the improvement of the outcomes of at least one production sector (Question 4) are mentioned in 90% of the NBSAPs, but in only 44% of the NBSAPs this relationship is identified for more than 4 sectors. Agriculture is the sector mentioned most often (in 63% of reports). For example, Belgium's NBSAP (p. 56) highlights that agricultural diversification can meet the demand for varied quality products, rural recreation activities as well as increases in farmers' profitability. The positive impact that biodiversity conservation can have on the tourism industry are also mentioned frequently (61% of reports), particularly in the Americas. For example, Mexico's NBSAP (p. 60) recognizes that tourism has great potential, considering the country's biological and cultural diversity. It also states that, with proper planning, promotion and development, the conservation of natural ecosystems can be reconciled with the economic and social needs of the population. Successful examples of such tourism include visits to the Monarch Butterfly, Calakmul and Montes Azules biosphere reserves and the protected community areas in Oaxaca. Sustainable management is also frequently recognized as benefitting fisheries (55% reports) and forestry (49% reports). For example, in Sudan's NBSAP (p. 55), it is mentioned that a sustainable and efficient use of the forest resources can contribute to biodiversity conservation, but also rural development and poverty alleviation. Overall, the way in which NBSAPs consider the potential benefits for sectors resulting from sustainable management and biodiversity conservation is fairly consistent across geographic regions and economic sectors (Table 3).

**Table 3:**
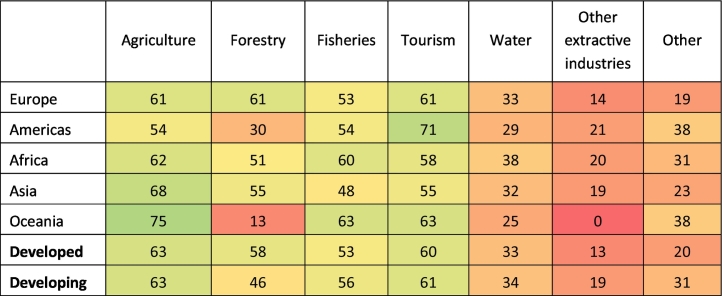
Percentage of NBSAPs, within each geographic region and economic group that discuss whether sustainable management plans (and biodiversity conservation) can improve the outcomes of each sector (Question 4). The colors are on a scale of red (0%), through yellow (50%) to green (100%).

The identification of conflicts and tradeoffs between biodiversity conservation and the output of production sectors (Question 5) is present in 50% of the NBSAPs. However, only 7% of the NBSAPs did so in four or more production sectors and it is therefore less commonly discussed than the perceived benefits of biodiversity. Agriculture is the sector most commonly mentioned, followed by forestry and other. The ‘other’ category includes, for example, infrastructures and transportation as mentioned in Italy's NBSAP (p. 77) or conflicts between biodiversity conservation and the development of renewable energy sources as mentioned in Croatia's NBSAP (p. 7). There were differences among the reports in this regard, with a higher proportion of developed nations identifying conflicts between biodiversity conservation and the production sectors, particularly agriculture and forestry (Table 4). For example, in Scotland's NBSAP it is recognized that, in upland areas, there are conflicting demands for livestock grazing, forestry, field sports, renewable energy developments, recreation and peatland restoration. Developing nations were more likely to identify conflicts with ‘other extractive industries’, for example, Sudan's NBSAP (p. 75) identifies the conflicts between mining and protected areas.

**Table 4:**
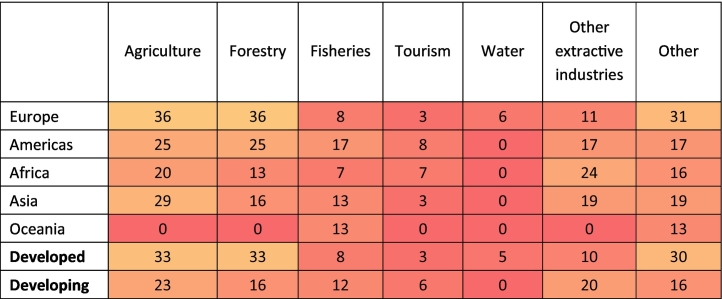
Percentage of NBSAPs, within each geographic region and economic group that identify conflicts and tradeoffs between biodiversity conservation and the output of production sectors (Question 5). The colors are on a scale of red (0%), through yellow (50%) to green (100%).

## 4 Discussion

This review of NBSAPs has shown that developing countries, particularly those in Africa, are - within their NBSAPs - more aware of the importance of mainstreaming biodiversity across economic sectors than developed countries, as seen through their higher normalized scores. This higher awareness is probably partly linked to the involvement of a broad range of stakeholders in the NBSAP development process, as developing countries were more likely to involve a greater range of stakeholders than developed countries. It is known that the involvement of several societal groups is key for the success of biodiversity mainstreaming (Huntley and Redford, 2014; SCBD, 2007). An inclusive process creates a sense of ownership that is likely to lead to increased commitment on the part all the stakeholders involved, as well as raising awareness of biodiversity values (Huntley and Redford, 2014; SCBD, 2007). This is all the more relevant considering that the economic sectors responsible for biodiversity loss, such as agriculture and forestry, are managed by a broad set of actors, beyond national governments (Karlsson-Vinkhuyzen et al., 2018). Those sectors have together up until 2010 caused almost 60% of the total reduction in global terrestrial biodiversity, and 55% of the expected loss up until 2050 (Kok et al., 2018), which highlights the imperative to engage all the actors involved in their governance in biodiversity strategic action plans. Our results, in accordance with Pisupati and Prip (2015), show that the majority of NBSAPs focussed on consultations with government sectors and agencies. There is still progress to be made in many countries, particularly those in Europe, regarding stakeholder involvement in the development of the NBSAPs, especially with the private sector and indigenous or local people.

Developing nations are also more likely to give specific details about the monetary contribution of biodiversity and ecosystem services to their economy in their national strategy, whilst a higher proportion of developed nations made no acknowledgement of this contribution. This is potentially due to the higher reliance of developing economies on agriculture and other primary production sectors (Gylfason, 2001). Developed countries also potentially have a greater disconnect with biodiversity and ecosystem services as they are more reliant on those from other countries (for example, Schmidt et al., 2009; Martín-López et al., 2018). The (developed) countries of Europe do, however, identify the loss of biodiversity as a threat to tourism. Our review of NBSAPs is therefore reflecting where countries perceive the (short-term) economic advantages of biodiversity and this does also suggest that the value of biodiversity as a support for ecosystem services is not being fully appreciated.

Note that our analysis of mainstreaming at the level of NBSAPs did not cover aspects of integration of concrete actions at the corporate level within different economic sectors. NBSAPs are political intentions of governments, and might draw an optimistic picture. They do not, for instance, include critical voices of a political opposition. Moreover, a political plan is only a nonbinding declaration of intent, and does not allow for an evaluation of actual implementation, i.e. mainstreaming in economic sectors and corporations. Indeed, evidence suggests that NBSAPs are a rather weak policy instrument and a minority have been endorsed across governments, whilst the majority have been adopted only within relevant environmental ministries (CBD, 2018a; UNEP, 2018). The mention of biodiversity mainstreaming within NBSAPs is often broad and does not specify the institutional and legal requirements that are needed to achieve the aspirational objectives. This factor, combined with a lack of a coordinating mechanism, creates a significant barrier to implementation (Prip, 2010; UNEP, 2018). An analysis of the NBSAP process within Finland identified a responsibility gap between the environmental administration and other policy sectors, which acts as a further barrier to the implementation of mainstreaming. They suggest that further developing the responsiveness of policy makers and enhancing institutional liability and accountability would help to close this gap (Sarkki et al., 2016). Additionally, there are concerns that mainstreaming biodiversity could be used by sectors for marketing purposes whilst destructive activities of a sector might (continue to) cause overall harm to biodiversity (Rodríguez-Labajos and Martínez-Alier, 2012). Moreover, there are critical concerns about the utilitarian framing used when expressing the economic value of biodiversity and the ecosystem services it provides to the economy (e.g. Adams, 2014; Schröter et al., 2014). An overly economic language might narrow down the discourse on biodiversity conservation, whilst a plurality of values with respect to biodiversity might be needed for successfully protecting biodiversity (Spash and Aslaksen, 2015).

It is also important to acknowledge that developing nations may achieve higher normalized scores because they more strictly adhere to the CBD guidance for producing NBSAPs, potentially as a result of the financial support available for the process. In order to reach a greater understanding of exactly why developing countries are achieving higher scores, a follow up analysis of how countries are de facto including biodiversity in sectoral policy plans and legislative change would be greatly beneficial. If countries score highly in both analyses, it would show that the appreciation of biodiversity mainstreaming in NBSAPs is indeed translating to positive action and output.

## 5 Conclusions

In conclusion, this review has shown that the foundations for mainstreaming biodiversity are being laid in the NBSAPs of many countries. However, we have highlighted that developed nations need to do more to acknowledge the value of biodiversity to their production sectors. The higher normalized scores achieved by developing nations suggests a greater connection of these countries to nature and a higher awareness of the interdependence of their economy with biodiversity and ecosystem services. In all countries there is evidently a greater need for biodiversity concerns to be included within the management plans of economic sectors, as this would offer a path to a future where mainstreaming is achieved on a global scale.

## Conflicts of interest

The authors declare no conflict of interest.

## Appendix A Supplementary data

Supplementary tables Image 1
