# Supplementary material for: Mainstreaming biodiversity: A review of national strategies
Source: Biol Conserv. 2019 Jul;235:157–63. doi: 10.1016/j.biocon.2019.04.016 (PMC7083249; doi:10.1016/j.biocon.2019.04.016)
Supplement: Supplementary file 1 — Supplementary tables [file mmc1.docx]

**Supplementary Information**

**Mainstreaming biodiversity: a review of national strategies**

Table S1 – Questions and keywords used to screen the NBSAPs. The keywords are given in English, Spanish (palabras clave) and French (mots clefs). * indicates truncated word.

| Question | | Keywords | Palabras Clave | Mots clefs |
| --- | --- | --- | --- | --- |
| 1. Which actors have been involved in the development of the NBSAP? | Public | Minist*  Agenc*  Universit*  Process  Development  Participant*  Actor(s)  Non-state actor(s) Stakeholders | Minister*  Agenc*  Universida*  Processo  Desenvolvimento  Participantes  Actor(es) | Minist*  Agenc*  Universit*  Processus  Développement  Participant*  Acteur(s)  Acteur(s) non-gouvernement* |
|  | Private |  |  |  |
|  | Civil Society |  |  |  |
|  | Other |  |  |  |
| 2. Are there references to the (potential) contribution of biodiversity/ecosystem services to the national economy?  (Yes, specific – 2; Yes, vague and general – 1; No – 0) |  | Mone* Valu* GDP (Gross Domestic Product) Income Employment Jobs Econ* | Monetario  Dinero  Valor  PIB (Producto Interno Bruto)  Ingresos  Empleo  Trabajo  Economia | Monét*  Valeur  PIB (Produit intérieur brut)  Revenu  Emploi  Jobs?  Econ* |
| 3. Is it discussed if biodiversity loss threatens the outcomes of particular sectors?  (Yes – 1; No - 0) | Agriculture | Loss  Outcome  Sector  Forest*  Agriculture  Tourism  Industr*  Deteriorat*  Damage  Affect*  Threat  Consequence  Product*  Resource  Risk  Water  Hydrol*  Hunt*  Fish* | Perdida  Resultado  Sector  Bosque  Agricultura  Turismo  Industria  Deterioro  Dano  Afectado  En peligro, amenaza  Consecuencia  Producto  Recurso  Riesgo  Agua  Hidrol*  Caza  Pesqueria | Perte  Revenu  Secteur  Forest  Forêt  Agricult*  Touris*  Industr*  Détérior*  Dommage  Menace  Conséquence  Product*  Ressource  Risque  Eaux  Hydrol*  Chasse  Pêche  Halieutique |
|  | Forestry |  |  |  |
|  | Fisheries |  |  |  |
|  | Tourism |  |  |  |
|  | Water supply |  |  |  |
|  | Other extractive activities |  |  |  |
|  | Other |  |  |  |
| 4. Is it discussed how sustainable management plans, (and biodiversity conservation) can contribute to the improvement of the outcomes of particular sectors?  (Yes – 1; No - 0) | Agriculture | Loss  Outcome  Sector  Forest*  Agriculture  Tourism  Industr*  Deteriorat*  Damage  Affect*  Threat  Consequence  Product*  Resource  Risk  Water  Hydrol*  Hunt*  Fish*  Sustainable Management  Certificat*  Label  Benefi*  Improve | Perdida  Resultado  Sector  Bosque  Agricultura  Turismo  Industria  Deterioro  Dano  Afectado  En peligro, amenaza  Consecuencia  Producto  Recurso  Riesgo  Agua  Hidrol*  Caza  Pesqueria  Sostenible  Manejo  Certifica*  Sello  Beneficio  Mejoramento | Perte  Revenu  Secteur  Forest  Forêt  Agricult*  Touris*  Industr*  Détérior*  Dommage  Menace  Conséquence  Product*  Ressource  Risque  Eaux  Hydrol*  Chasse  Pêche  Halieutique  Durable  Gestion  Label  Bénéfic*  Amélior* |
|  | Forestry |  |  |  |
|  | Fisheries |  |  |  |
|  | Tourism |  |  |  |
|  | Water supply |  |  |  |
|  | Other extractive activities |  |  |  |
|  | Other |  |  |  |
| 5. Is it discussed if biodiversity conservation threatens the outcomes of particular sectors?  (Yes – 1; No - 0) | Agriculture | Compensat*  Subsid*  Pay*  Conflict  Mitigat*  Income foregone  Voluntary  Grant  Scheme  Compatibility  Tension  Quota  Cost | Compensa*  Subsidios  Conflicto  Mitigacion  Amenaza al desarollo  Voluntario  Esquema  Compatible  Tensión  Cuota  Costo | Compens*  Subvention*  Paye*  Conflit  Atténue  Perte de revenu  Volontaire  Bourse  Schéma  Compatible  Tension  Quotat  Coût  Indemnis* |
|  | Forestry |  |  |  |
|  | Fisheries |  |  |  |
|  | Tourism |  |  |  |
|  | Water supply |  |  |  |
|  | Other extractive activities |  |  |  |
|  | Other |  |  |  |

Table S2 – Details of individual countries analyzed.

|  | Year received | Sub-region | Economic development | Normalized score |
| --- | --- | --- | --- | --- |
| **Africa** |  |  |  |  |
| Algeria | 2016 | Northern Africa | Developing | 2.08 |
| Egypt | 2016 | Northern Africa | Developing | 2.33 |
| Morocco | 2016 | Northern Africa | Developing | 3.00 |
| Sudan | 2015 | Northern Africa | Developing | 2.75 |
| Burundi | 2015 | Eastern Africa | Developing | 3.67 |
| Comoros | 2016 | Eastern Africa | Developing | 2.42 |
| Djibouti | 2017 | Eastern Africa | Developing | 3.00 |
| Eritrea | 2015 | Eastern Africa | Developing | 2.00 |
| Ethiopia | 2016 | Eastern Africa | Developing | 2.83 |
| Madagascar | 2016 | Eastern Africa | Developing | 2.42 |
| Malawi | 2016 | Eastern Africa | Developing | 2.58 |
| Mauritius | 2017 | Eastern Africa | Developing | 2.92 |
| Mozambique | 2016 | Eastern Africa | Developing | 1.75 |
| Rwanda | 2017 | Eastern Africa | Developing | 3.67 |
| Seychelles | 2014 | Eastern Africa | Developing | 3.75 |
| Somalia | 2016 | Eastern Africa | Developing | 2.00 |
| Uganda | 2015 | Eastern Africa | Developing | 3.33 |
| United Republic of Tanzania | 2016 | Eastern Africa | Developing | 2.92 |
| Zambia | 2015 | Eastern Africa | Developing | 2.17 |
| Zimbabwe | 2016 | Eastern Africa | Developing | 2.75 |
| Cameroon | 2014 | Middle Africa | Developing | 4.00 |
| Chad | 2016 | Middle Africa | Developing | 1.58 |
| Congo | 2015 | Middle Africa | Developing | 2.25 |
| Democratic Republic of the Congo | 2016 | Middle Africa | Developing | 2.58 |
| Equatorial Guinea | 2015 | Middle Africa | Developing | 3.75 |
| Sao Tome & Principe | 2016 | Middle Africa | Developing | 2.67 |
| Botswana | 2016 | Southern Africa | Developing | 1.83 |
| Namibia | 2015 | Southern Africa | Developing | 4.50 |
| South Africa | 2016 | Southern Africa | Developing | 3.00 |
| Swaziland | 2017 | Southern Africa | Developing | 2.00 |
| Benin | 2016 | Western Africa | Developing | 2.58 |
| Burkina Faso | 2011 | Western Africa | Developing | 1.92 |
| Cabo Verde | 2016 | Western Africa | Developing | 3.50 |
| Côte d’Ivoire | 2016 | Western Africa | Developing | 2.25 |
| Ghana | 2017 | Western Africa | Developing | 2.50 |
| Guinea | 2016 | Western Africa | Developing | 2.00 |
| Guinea-Bissau | 2016 | Western Africa | Developing | 2.75 |
| Liberia | 2017 | Western Africa | Developing | 2.92 |
| Mali | 2015 | Western Africa | Developing | 3.17 |
| Mauritania | 2014 | Western Africa | Developing | 2.92 |
| Niger | 2015 | Western Africa | Developing | 3.33 |
| Nigeria | 2015 | Western Africa | Developing | 2.67 |
| Senegal | 2015 | Western Africa | Developing | 3.50 |
| The Gambia | 2016 | Western Africa | Developing | 2.83 |
| Togo | 2015 | Western Africa | Developing | 2.75 |
| **Americas** |  |  |  |  |
| Canada | 2016 | Northern America | Developed | 0.75 |
| Antigua and Barbuda | 2014 | Caribbean | Developing | 2.83 |
| Cuba | 2016 | Caribbean | Developing | 0.75 |
| Dominica | 2014 | Caribbean | Developing | 2.42 |
| Dominican Republic | 2012 | Caribbean | Developing | 3.67 |
| Grenada | 2016 | Caribbean | Developing | 1.00 |
| Jamaica | 2016 | Caribbean | Developing | 2.75 |
| St. Kitts & Nevis | 2016 | Caribbean | Developing | 1.75 |
| Belize | 2016 | Central America | Developing | 3.50 |
| Costa Rica | 2017 | Central America | Developing | 3.50 |
| El Salvador | 2014 | Central America | Developing | 3.25 |
| Guatemala | 2014 | Central America | Developing | 3.17 |
| Mexico | 2016 | Central America | Developing | 2.83 |
| Nicaragua | 2016 | Central America | Developing | 2.00 |
| Argentina | 2017 | South America | Developing | 3.50 |
| Brazil | 2016 | South America | Developing | 1.83 |
| Colombia | 2012 | South America | Developing | 3.67 |
| Ecuador | 2016 | South America | Developing | 2.17 |
| Guyana | 2015 | South America | Developing | 3.33 |
| Paraguay | 2016 | South America | Developing | 2.33 |
| Peru | 2015 | South America | Developing | 3.83 |
| Suriname | 2013 | South America | Developing | 3.83 |
| Uruguay | 2016 | South America | Developing | 2.33 |
| Venezuela | 2011 | South America | Developing | 2.83 |
| **Asia** |  |  |  |  |
| Kyrgyzstan | 2016 | Central Asia | Developing | 1.08 |
| China | 2010 | Eastern Asia | Developing | 0.92 |
| DPR Korea | 2007 | Eastern Asia | Developing | 2.42 |
| Japan | 2013 | Eastern Asia | Developed | 2.25 |
| Mongolia | 2015 | Eastern Asia | Developing | 2.25 |
| Republic of Korea | 2014 | Eastern Asia | Developing | 2.67 |
| Brunei Darussalam | 2015 | South-eastern Asia | Developing | 1.08 |
| Cambodia | 2016 | South-eastern Asia | Developing | 3.33 |
| Lao PDR | 2016 | South-eastern Asia | Developing | 3.33 |
| Malaysia | 2016 | South-eastern Asia | Developing | 2.25 |
| Myanmar | 2012 | South-Eastern Asia | Developing | 3.92 |
| Philippines | 2016 | South-eastern Asia | Developing | 3.00 |
| Thailand | 2016 | South-eastern Asia | Developing | 2.17 |
| Timor-Leste | 2011 | South-Eastern Asia | Developing | 3.83 |
| Viet Nam | 2015 | South-eastern Asia | Developing | 3.17 |
| Afghanistan | 2014 | Southern Asia | Developing | 2.25 |
| Bangladesh | 2016 | Southern Asia | Developing | 2.67 |
| Bhutan | 2014 | Southern Asia | Developing | 2.50 |
| India | 2014 | Southern Asia | Developing | 2.92 |
| Iran | 2016 | Southern Asia | Developing | 0.50 |
| Maldives | 2016 | Southern Asia | Developing | 3.00 |
| Nepal | 2014 | Southern Asia | Developing | 2.67 |
| Sri Lanka | 2016 | Southern Asia | Developing | 3.17 |
| Armenia | 2015 | Western Asia | Developing | 1.58 |
| Azerbaijan | 2016 | Western Asia | Developing | 0.58 |
| Bahrain | 2016 | Western Asia | Developing | 2.42 |
| Georgia | 2014 | Western Asia | Developing | 3.42 |
| Iraq | 2016 | Western Asia | Developing | 1.83 |
| Jordan | 2015 | Western Asia | Developing | 1.58 |
| Lebanon | 2016 | Western Asia | Developing | 3.08 |
| Qatar | 2016 | Western Asia | Developing | 1.25 |
| **Europe** |  |  |  |  |
| Belarus | 2010 | Eastern Europe | Developed | 1.25 |
| Czech Republic | 2016 | Eastern Europe | Developed | 2.08 |
| Hungary | 2015 | Eastern Europe | Developed | 2.00 |
| Poland | 2015 | Eastern Europe | Developed | 0.75 |
| Republic of Moldova | 2015 | Eastern Europe | Developed | 2.58 |
| Romania | 2016 | Eastern Europe | Developed | 1.75 |
| Russian Federation | 2015 | Eastern Europe | Developed | 3.25 |
| Slovakia | 2015 | Eastern Europe | Developed | 1.42 |
| Ukraine | 2016 | Eastern Europe | Developed | 0.92 |
| Denmark | 2015 | Northern Europe | Developed | 3.25 |
| England | 2011 | Northern Europe | Developed | 2.75 |
| Estonia | 2014 | Northern Europe | Developed | 2.33 |
| Finland | 2013 | Northern Europe | Developed | 3.50 |
| Ireland | 2012 | Northern Europe | Developed | 3.00 |
| Lithuania | 2017 | Northern Europe | Developed | 1.25 |
| Scotland | 2013 | Northern Europe | Developed | 3.75 |
| Sweden | 2016 | Northern Europe | Developed | 1.08 |
| Albania | 2016 | Southern Europe | Developed | 1.92 |
| Andorra | 2016 | Southern Europe | Developed | 1.17 |
| Bosnia and Herzegovina | 2016 | Southern Europe | Developed | 1.67 |
| Croatia | 2017 | Southern Europe | Developed | 1.92 |
| Greece | 2014 | Southern Europe | Developed | 1.67 |
| Italy | 2010 | Southern Europe | Developed | 3.92 |
| Malta | 2012 | Southern Europe | Developed | 1.67 |
| Montenegro | 2017 | Southern Europe | Developed | 1.92 |
| Serbia | 2011 | Southern Europe | Developed | 2.33 |
| Spain | 2012 | Southern Europe | Developed | 2.50 |
| Austria | 2015 | Western Europe | Developed | 3.58 |
| Belgium | 2014 | Western Europe | Developed | 2.25 |
| France | 2011 | Western Europe | Developed | 2.75 |
| Germany | 2016 | Western Europe | Developed | 0.42 |
| Liechtenstein | 2014 | Western Europe | Developed | 0.50 |
| Luxembourg | 2017 | Western Europe | Developed | 1.42 |
| Netherlands | 2014 | Western Europe | Developed | 2.75 |
| Switzerland | 2012 | Western Europe | Developed | 3.08 |
| European Union | 2011 |  | Developed | 3.08 |
| **Oceania** |  |  |  |  |
| Australia | 2011 | Australia and New Zealand | Developed | 1.25 |
| New Zealand | 2016 | Australia and New Zealand | Developed | 1.58 |
| Solomon Islands | 2016 | Melanesia | Developing | 2.83 |
| Kiribati | 2017 | Micronesia | Developing | 2.33 |
| Nauru | 2016 | Micronesia | Developing | 1.58 |
| Niue | 2015 | Polynesia | Developing | 1.25 |
| Samoa | 2016 | Polynesia | Developing | 2.25 |
| Tuvalu | 2014 | Polynesia | Developing | 1.92 |
